# Paradoxes: Cholesterol and Hypoxia in Preeclampsia

**DOI:** 10.3390/biom14060691

**Published:** 2024-06-13

**Authors:** Nancy R. Hart

**Affiliations:** PeaceHealth St. Joseph Medical Center, Bellingham, WA 98225, USA; kynrhart@gmail.com

**Keywords:** preeclampsia, hypoxia inducible factors, cholesterol, dietary lipids, Hedgehog (Hh), Wnt/βcatenin, membrane biophysics, endothelial nitric oxide synthase, dyslipidemia, oxidative stress

## Abstract

Preeclampsia, a hypertensive disease of pregnancy of unknown etiology, is intensely studied as a model of cardiovascular disease (CVD) not only due to multiple shared pathologic elements but also because changes that develop over decades in CVD appear and resolve within days in preeclampsia. Those affected by preeclampsia and their offspring experience increased lifetime risks of CVD. At the systemic level, preeclampsia is characterized by increased cellular, membrane, and blood levels of cholesterol; however, cholesterol-dependent signaling, such as canonical Wnt/βcatenin, Hedgehog, and endothelial nitric oxide synthase, is downregulated indicating a cholesterol deficit with the upregulation of cholesterol synthesis and efflux. Hypoxia-related signaling in preeclampsia also appears to be paradoxical with increased Hypoxia-Inducible Factors in the placenta but measurably increased oxygen in maternal blood in placental villous spaces. This review addresses the molecular mechanisms by which excessive systemic cholesterol and deficient cholesterol-dependent signaling may arise from the effects of dietary lipid variance and environmental membrane modifiers causing the cellular hypoxia that characterizes preeclampsia.

## 1. Introduction

Preeclampsia, a leading cause of maternal and fetal morbidity and mortality worldwide, affects 3–8% of pregnancies and causes about 76,000 maternal and 500,000 fetal and neonatal deaths yearly [1]. Clinically, preeclampsia is recognized as the new onset of hypertension after 20 weeks of gestational age, usually associated with proteinuria and edema. The maternal endothelium is integral to preeclampsia pathophysiology with reduced endothelial nitric oxide synthase activity, impaired vasodilation, and increased sensitivity to vasoconstrictors, such as angiotensin I. Other basic pathophysiologic elements of preeclampsia include dyslipidemia, cellular hypoxia, oxidative stress, inflammation, immune dysfunction, increased ceramides, abnormal membrane rafts, autoantibodies, and protein misfolding. Although aspirin delays the onset of preeclampsia, the underlying molecular cause is unknown, so no cure is currently available other than delivery of the baby and placenta, sometimes quite prematurely. In a matter of weeks, pregnant people with no known disease in early pregnancy may progress to multi-organ failure with a pathology identical to end-stage hypertensive disease with severe metabolic derangements, such as proteinuria, abnormal liver functions, thrombocytopenia, loss of vision, diffuse intravascular coagulation, seizures, and cerebral edema in the mother and growth restriction, prematurity, and death in the fetus. Although maternal metabolic dysregulation generally resolves within days after delivery of the placenta, those with preeclampsia and their offspring experience an increased lifetime risk of cardiovascular disease [2].

Lipids are intimately involved in preeclampsia pathology with an increased risk for those with increased serum trans (TUFAs) [3] or polyunsaturated fatty acids (PUFAs) [4], reduced serum omega-3 PUFAs [5], or dyslipidemia [6,7]; metabolomic studies consistently show sterol, phospholipid, and sphingolipid metabolism as major discriminatory pathways [8,9,10,11,12,13,14,15,16]. However, interventional studies of the effects of diet on preeclampsia show inconsistent findings. For example, weight reduction [17] and a reduction in dietary TUFAs significantly reduce the risk [18], yet adherence to a vegan diet increases the risk [19]. Many studies show an increased risk of preeclampsia with increased dietary PUFAs [20], but other populations show a reduced risk with PUFA supplementation [21]. The role of cholesterol in the etiology of human preeclampsia is especially confusing given evidence of systemic cholesterol excess alongside deficits in cholesterol-dependent signaling pathways. Standard medical advice is to reduce dietary cholesterol, yet some studies show that those with higher cholesterol intake have a reduced preeclampsia risk [22].

An animal model of preeclampsia (Reduced Uterine Perfusion Pressure or RUPP) can be created by partially occluding vessels that supply the pregnant uterus. This ischemic model reproduces many of the clinical features of human preeclampsia, including increased blood pressure and total peripheral resistance with impaired nitric oxide-dependent vasodilation, impaired renal glomerular filtration with proteinuria, a reduced ejection fraction and cardiac output, immune dysfunction with increased prehypertensive cytokines, T cells, and B cells, and increased capillary permeability with cerebral edema and a reduction in blood–brain barrier integrity [23]. In laboratory animals, these clinical features may be traced directly to the effects of hypoxia-related signaling and are mitigated by inhibiting Hypoxia Inducible Factor-2α (HIF-2α) [24]. Human preeclampsia is also characterized by increased HIF signaling [25]. Intriguingly, in humans with early onset preeclampsia (EOPE), defined as onset prior to 34 weeks of gestational age, measurements of oxygen concentrations in the villous space where maternal blood contacts the placental villous trophoblast and in the uterine vein leaving the maternal uterus do not show hypoxia but higher partial pressures of oxygen than in normal healthy pregnancies. However, in these pregnancies, fetal oxygenation is reduced [26], indicating that the movement of oxygen across the placenta is impaired with EOPE. Because cholesterol presents a major impediment to the diffusion of oxygen across membranes, elevated membrane cholesterol offers a mechanism for increased hypoxia-related signaling from the placenta. However, the fundamental cause of the increased membrane cholesterol and reduced movement of oxygen across the placenta is unknown.

Over the last century, dietary lipid intake in the United States has markedly changed and environmental toxins, such as perfluoroalkyl substances (PFAS), bisphenol A (BPA), and inhaled hydrocarbons, have increased. This review first outlines the effects of dietary lipids and environmental membrane-modifying agents on membrane biophysics, cholesterol sequestration, and oxygen diffusion and then integrates these findings with observational and interventional studies in those with preeclampsia. This integration provides a molecular mechanism for preeclampsia’s hypoxia and aberrant cholesterol signaling.

## 2. Changes in Dietary Lipids in the United States of America (US) over the Last Century

During the last century, the US population has undergone a widespread uncontrolled trial of the effects of dietary lipids on human health. From 1909 until 2011, the US Department of Agriculture (USDA) maintained data on foods consumed in the US each year. Over this interval, the per capita daily consumption of saturated fats **decreased** from 28 pounds in 1912 to 13.4 pounds in 2011, while the consumption of PUFAs in vegetable oils **increased** from 11.3 to 64.5 pounds. The estimated per capita consumption of soybean oil, now the most used cooking oil in the US, increased over 1000-fold from 1909 to 1999 [27]. Total fat intake increased from 41 to 79 pounds per capita per year, an increase almost totally due to an increase in PUFAs [28,29]. Over the last century, worldwide TUFA consumption rose from an optimal level of less than 1% of daily dietary fats to a high of 6.5% in some countries [30,31]. Although levels of TUFA consumption in packaged foods fell after 2013 when the USDA classified TUFAs as a food additive [32] and the 2018 World Health Organization initiative limited dietary intake [33], TUFAs form at a rate of about 3.5 g per 100 g of polyunsaturated oil with each cooking episode [34]. The reuse of oil in deep fryers and the use of spent oil in livestock feeds means that there is an ongoing yet unmeasured supply of TUFAs in human diets. Humans evolved with an omega 6:3 ratio of about 1:1, while Western diets now average a ratio of 16:1 with levels rising to 50:1 in areas of urban India [35]. Archeologic studies estimate daily cholesterol intake of our hunter/gatherer genetic ancestors at 480 to 880 mg per day [36,37], yet average daily dietary cholesterol in the United States in 2014 was 293 mg per day [38].

In summary, lipids in modern diets are characterized by (1) a marked increase in dietary fat almost wholly from polyunsaturated vegetable oils (increased unsaturation index), (2) a high omega 6:3 ratio, (3) increased trans fatty acids, (4) reduced dietary cholesterol, and (5) reduced saturated fatty acids.

Because dietary fats are rapidly incorporated into tissues, these dietary variances may predictably change the biophysical and signaling properties of placental and maternal membranes in preeclampsia.

## 3. Environmental Lipids and Membrane Modifiers Affect Preeclampsia Risk

Despite some inconsistency [39], environmental studies generally demonstrate an increased risk of preeclampsia for those with chronic exposure to air pollution [40,41], traffic-related air pollution [42,43], indoor air pollution [44,45], and inhaled volatile organic compounds [46,47]. Exposure to only non-hydrocarbon particulate air pollution is not clearly associated with an increased preeclampsia risk [48,49]. Specific components of fine particulate air pollution differentially increase preeclampsia risk, with organic carbon components having a greater adverse effect [50]. It appears that air pollution containing pollutants, such as benzene, toluene, xylene (volatile organic compounds), and oil, tar, gasoline, and diesel combustion components that may be absorbed through the lung alveolar surface, may be sources of an increased preeclampsia risk. These compounds modify cell membrane fluidity and other biophysical and biochemical membrane properties [51] and are associated with the clinical symptoms of increased susceptibility to vasospasm, increased blood pressure, and increased atherosclerotic lesions. They also dysregulate cellular signaling and gene expression [52].

Agents, such as PFAS and polychlorinated biphenyls (PCBs), man-made chemicals that are widely distributed in the environment, may modify the biophysical properties of cell membranes and lipid metabolism [53] and are associated with increased risk of preeclampsia [54,55,56,57].

## 4. Placental Hypoxia and Hypoxia-Inducible Factors (HIF)

The term “hypoxia” is often used in describing preeclampsia pathology partially due to the success of the RUPP animal model, which uses ischemia to create preeclampsia-like pathology, but also due to the marked increase in HIFs in placentae of human preeclampsia [58]. The regulation of HIF is complex. With normal oxygen concentrations in the placental and endothelial cytoplasm, HIFs are continuously produced but rapidly degraded through the action of prolyl hydroxylase domains. However, at low oxygen tension, prolyl hydroxylation is suppressed causing the stabilization of HIFs, which translocate to the nucleus and stimulate the transcription of over 60 hypoxia-responsive genes regulating diverse pathways, such as fibrosis [59], angiogenesis, erythropoiesis, and mitochondrial homeostasis [60]. The dysregulation of these pathways with the elevation of HIFs is central to human preeclampsia pathophysiology [61]. Although HIFs may also be stimulated under normal oxygen conditions by other factors, such as heat shock [62], increased mechanical stretch [63], reactive oxygen species [64], growth stimuli [65], thiamine deficiency [66], inflammation [67], and hyperglycemia [68,69], a significant part of preeclampsia’s elevated HIFs likely arises from placental cellular hypoxia [70].

Low oxygen tension also stimulates changes in cholesterol homeostasis in human trophoblasts with increases in cholesterol synthesis and efflux to the maternal serum, changes seen in human preeclampsia [71,72]. **Thus, cellular hypoxia may contribute both to the increase in HIF signaling and changes in cholesterol homeostasis, which characterize preeclampsia**. However, the origin of placental and endothelial cell hypoxia remains a mystery.

Early animal models of preeclampsia show that dietary changes reduce the placental uptake of oxygen. A 1965 study with Columbia-Sherman rats investigated the effects of an experimental diet deficient in vitamin E to which 5% oxidized cod liver oil is added. With this diet, the transport of sodium, potassium, and oxygen in the placenta is reduced [73]. Although HIF would not be discovered for almost thirty years [74], presumably a reduction in oxygen uptake by the placenta would have resulted in placental cell hypoxia and elevated HIFs in these animals.

Early placental development in humans requires a hypoxic environment. In this environment, HIF-1α and Transforming Growth Factor β3, which inhibits trophoblastic differentiation, are increased. In healthy pregnancies, toward the end of the first trimester, spiral artery remodeling results in an increase in villous blood flow and a reduction in both HIF-1α and TGFβ3, stimulating normal placental development. However, in preeclampsia, the normal increase in villous blood flow does not occur, hypoxia continues with high levels of HIF and TGFβ3, and placental development is impaired [75].

These studies raise two fundamental questions about preeclampsia pathology: “What impedes movement of oxygen across the placenta?” and “What impairs placental development causing reduction in late first trimester villous blood flow?” We will first address the mechanism by which dietary and environmental elements may induce membrane changes, which impede transplacental oxygen diffusion, and then consider the role that these molecules play in reducing cholesterol-dependent angiogenic signaling and creating poor placentation.

## 5. Impedance to Transmembrane Oxygen Diffusion

The availability of oxygen in fetal tissues depends on three elements: oxygen saturation in the maternal blood, the exposure time of maternal RBCs to the villus trophoblast, and the transmembrane oxygen diffusion time [76,77]. As mentioned above, in EOPE, maternal blood in the villous space contains more, not less, oxygen. Although hypertension and hemodynamic changes may alter the exposure time of RBCs at the trophoblast, hypoxia and reduced villous blood flow precede the onset of these changes by weeks with studies of placental exchange showing a decrease in oxygen conductance at the level of the villus trophoblast and fetal vasculature [78]. We will focus on the third element, that is, characteristics of the placental and fetal membranes, which may reduce the movement of oxygen from maternal red blood cells across the placenta to fetal blood.

Studies of in vitro artificial membranes and ex vivo natural membranes show that a variety of changes in membranes reduce trans-membrane oxygen diffusion: unsaturation of membrane lipids, increased cytoskeletal elements, increased membrane stiffness, increased cholesterol, increased thickness, and increased protein content [79].

The role of membrane cholesterol in reducing the trans-membrane movement of oxygen is well studied in ophthalmology where cholesterol protects the highly oxidizable light-sensing molecule rhodopsin and its surrounding PUFAs from oxidative damage. Cholesterol’s effect may also be seen in the lens of the eye, which is surrounded by a membrane with a very high cholesterol content that protects the lens from oxidative damage and prevents cataract formation. As the cholesterol concentration in plasma membranes rises above the normal 10 to 20%, the membrane permeability coefficient (P_M_) for oxygen declines proportionally and the movement of oxygen across membranes may be reduced to almost zero [80,81,82]. At concentrations over about 50%, membrane saturation allows cholesterol/cholesterol bilayer domains to form. **These domains have a P_M_ of about one tenth that of surrounding membranes and present a significant barrier to oxygen transport** [79]. Dietary and environmental lipids are largely responsible for determining the cholesterol content and biophysical properties of endothelial and placental membranes.

## 6. Effects of Molecular Components on Membrane Biophysics and Phase Separation

In the laboratory, a phospholipid bilayer forms spontaneously if phospholipids are mixed with cholesterol in an aqueous environment. The physical characteristics of this bilayer membrane are determined by the component lipids. A variety of head groups, such as phosphatidylserine, phosphatidylethanolamine, phosphatidylcholine, sphingomyelin, and ceramide, each having one or two attached fatty acid tails, creates an enormous diversity of membrane lipids [83]. Each phospholipid or sphingolipid molecule differs in chemical and physical characteristics allowing properties, such as fluidity or packing density, charge, phase separation, and permeability, to be finely tuned for optimal membrane function. In general, longer fatty acids and fewer double bonds increase the membrane melting point, reduce membrane fluidity and permeability, and increase cholesterol affinity. Shorter fatty acids and more double bonds reduce packing density and cholesterol affinity resulting in a more fluid and permeable membrane. The oxidation of fatty acids and sterols generally reduces the packing density causing a more fluid lipid bilayer membrane. The membrane composition also influences the formation of cytoskeletal elements, stiffness, thickness, association of proteins, and formation of cholesterol crystals.

**Figure 1:**
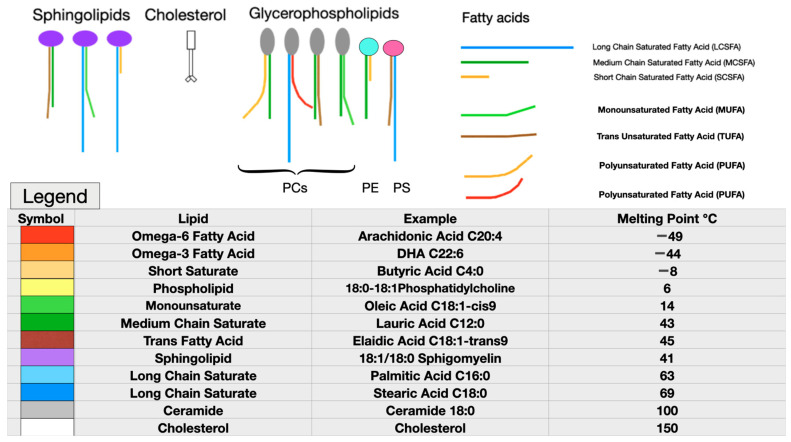
Representative membrane lipids. Note the very low melting points of the highly unsaturated omega-6 and omega-3 essential fatty acids, as well as the very-short-chain saturated butyric acid (red, bright orange, and yellow). Monounsaturated fatty acids, such as oleic acid, which is abundant in olive oil or medium-chain saturated fatty acids, such as lauric acid found in coconut oil, have moderate melting points (green). Long-chain saturated fatty acids (LCSFAs) with no double bonds, such as palmitic and stearic acids found in beef, have high melting points and high cholesterol affinity (purple, blue, and greys). Although cholesterol (white) has a very high melting point, due to its planar head and short tail, it acts as a plasticizer to maintain normal viscosity over a wide range of membrane compositions. Cholesterol also reduces membrane permeability to water. Phosphatidylcholine (PC), phosphatidylethanolamine (PE), and phosphatidylserine (PS) are pictured as representative glycerophospholipids (top left). Each phospholipid head group has two fatty acids attached, with the most usual arrangement being a saturated fatty acid at position one and an unsaturated fatty acid at position two [86]. (Parts of figure used with permission from Hart NR. A theoretical model of dietary lipid variance as the origin of primary ciliary dysfunction in preeclampsia [87]).

Even a small change in molecular structure may cause major differences in how a molecule behaves in a membrane. For example, elaidic and oleic acids have identical chemical formulae, each containing 18 carbons with one double bond at position nine. Oleic acid’s double bond in a cis configuration gives this fatty acid’s shape a distinct kink, which reduces the packing density, while increasing membrane fluidity and permeability, compared to elaidic acid’s trans double bond. This simple change in the configuration of one double bond gives elaidic acid a melting point 31℃ higher than that of oleic acid. **Elaidic acid also has a much higher cholesterol affinity, requiring eight times as much cholesterol as oleic acid to be incorporated into a membrane** [84]. Thus, substituting the trans fatty acid elaidic acid for oleic acid raises the membrane cholesterol concentration, a finding common to both CVD and preeclampsia [85]. **Figure 1** illustrates some representative fatty acids and headgroup molecules, which compose living cell membranes.

**Figure 2:**
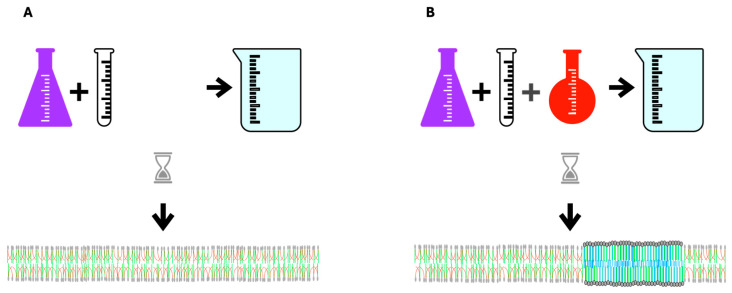
In vitro spontaneous lipid bilayer formation. (**A**) Upon mixing phospholipids with similar melting points and cholesterol in an aqueous medium, a homogeneous bilayer forms. (**B**) When a low-melting-point lipid is added to the mixture the lipids do not mix homogeneously allowing areas with greater and lesser order to form. PUFA’s aversion to cholesterol is the driving force in this process of phase separation in which areas of high-melting-point lipids gather with cholesterol away from low-melting-point PUFAs. The thicker, denser blue area in the middle of the bilayer membrane represents a liquid-ordered area, which corresponds to rafts in living cells. CVD is characterized by excess membrane rafts.

The high aversion of PUFAs for cholesterol drives a process called **phase separation** in which membrane lipids with diverse melting points separate into liquid-ordered and liquid-disordered areas or domains. Molecules with higher melting points and high cholesterol affinity, such as LCSFAs, group together to form liquid-ordered areas corresponding to **rafts** in living membranes with more fluid non-raft areas having lower melting point lipids. **The increased disparity of melting points in component lipids increases phase separation** **(Figure 2)**.

## 7. Dietary Challenges to Membrane Homeoviscosity

**Figure 3:**
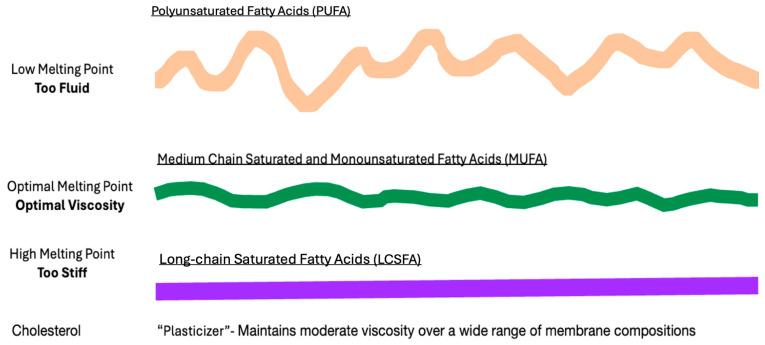
Dietary lipids and membrane viscosity. When the membrane homeostasis of laboratory animals is challenged with a high level of dietary low-melting-point PUFAs, fatty acids are rapidly incorporated into membranes, causing the bilayer to become excessively fluid (wavey orange line). To compensate, the increased synthesis and incorporation of cholesterol and high-melting-point LCSFAs increases the membrane rigidity (straight purple line), allowing membranes to regain normal viscosity (green moderately wavey line).

The importance of maintaining normal membrane viscosity for cell viability was first studied in single-cell organisms and poikilotherms by Sinensky in the 1970s. This work defined a mechanism by which cells responded to cold or heat stress through the metabolic adaptations of membrane composition to regain optimal membrane viscosity [88]. More recently, the Levental lab challenged membrane homeoviscosity in rats with a large dose of dietary PUFAs. Dietary lipids were rapidly incorporated into tissues with increased dietary PUFAs producing excessively fluid membranes with high permeability, similar to findings in heat stress. To maintain optimal membrane viscosity after the incorporation of highly unsaturated fatty acids, there is compensatory remodeling of the lipidome with the upregulation of saturated fatty acids and cholesterol synthesis, resulting in the restoration of normal membrane packing and permeability. **(Figure 3)** If this compensatory response is blocked, the cell dies, demonstrating the critical necessity of maintaining normal membrane viscosity for cell viability [89]. These findings raise the possibility that major changes in human dietary lipids affect membrane homeoviscosity and cause lipid remodeling.

## 8. Excessively Fluid Lipid Bilayers Increase Membrane Stiffness

**Figure 4:**
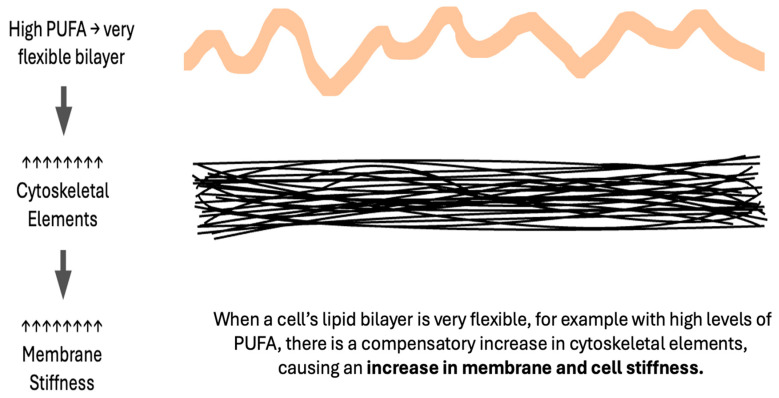
Excessively fluid bilayers cause compensatory increases in the cytoskeleton.

In considering the six factors, which impede the movement of oxygen across membranes (increased membrane unsaturation, cytoskeletal elements, stiffness, cholesterol, thickness, or proteins), an apparent inconsistency arises. An increase in the unsaturation index and in membrane stiffness both reduce oxygen diffusion, yet increased unsaturation increases **bilayer** fluidity. A similar inconsistency is seen in the preeclampsia literature with many lipidomic datasets indicating an increased unsaturation index [4,10,14,20], but measurably stiffer maternal RBC membranes [90,91,92] as is the case in non-pregnant individuals with chronic hypertension [93]. The key to this paradox is the role of excessively fluid lipid bilayers in stimulating a compensatory increase in cytoskeletal elements, producing a stiffer cell as measured via RBC deformability or atomic force microscopy [94,95,96,97]. **Thus, membrane stiffness is directly proportional to the membrane lipid unsaturation index (Figure 4).** The actions of magnesium, a medication commonly used to reduce seizure risk in women with preeclampsia, include a reduction in RBC stiffness [98] and the disassembly of cytoskeletal elements [99].

## 9. Melting Point Disparity, Membrane Rafts, and Cholesterol Content

In living cells, membrane rafts are defined as small, dynamic, sterol- and sphingolipid-enriched heterogeneous domains that compartmentalize cell membrane processes and serve as organizing centers for a complex network of signaling. Compared to the more fluid areas of the cell membrane, which are enriched in PUFAs with very low cholesterol affinity, rafts are enriched in LCSFAs with high cholesterol affinity; thus, rafts represent areas of increased cholesterol [100]. Lipid rafts roughly correspond to liquid-ordered phases in model membrane systems. In these systems, **greater disparity in the melting point of membrane lipids favors increased phase separation and raft formation, meaning that PUFAs with very low melting points are major driving forces for phase separation whilst monounsaturated fatty acids inhibit raft formation** [101]. Animal studies show increased PUFA results in changes in membrane lateral organization, producing larger rafts, which function poorly as signaling platforms [102]. **In human CVD, membrane rafts are more numerous and stable** [103], with lipidomic evidence of a similar increase in preeclampsia [104].

**Figure 5:**
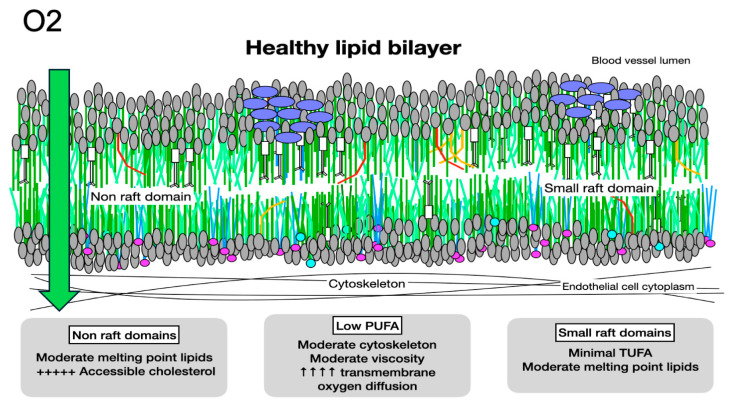
Oxygen diffusion in healthy membranes. Healthy membranes present almost no barrier to oxygen diffusion. With primarily moderate-melting-point lipids, moderate cytoskeletal elements, less than 1% PUFAs, small rafts, and minimal molecules with very high cholesterol affinity, such as LCSFAs and trans unsaturated fatty acids, the total cholesterol content of a healthy membrane is moderate. The cholesterol present is associated with molecules with low or moderate cholesterol affinity, making it accessible and available for signaling.

**Figure 6:**
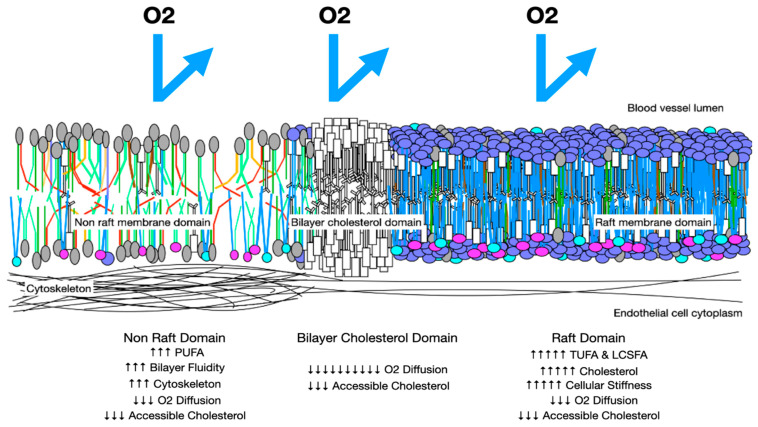
Oxygen diffusion in preeclampsia membranes. In preeclampsia, membranes have more rafts with increased cholesterol and LCSFAs, which increase membrane thickness and reduce trans-membrane oxygen diffusion. Areas with increased PUFAs may have increased cytoskeletal elements and increased stiffness. These factors reduce trans-membrane oxygen diffusion. Cholesterol/cholesterol bilayer areas reduce oxygen D_M_ to 10% that of a health membrane.

The segregation of lipids into raft areas and non-raft areas may create microdomains with features that reduce trans-membrane oxygen diffusion. The high unsaturation index, increased cytoskeletal elements, and stiffness of non-raft areas with a high PUFA content reduce oxygen diffusion. High cholesterol content, increased proteins, and high cholesterol affinity molecules, such as LCSFAs in raft areas also reduce oxygen diffusion. If the cholesterol content of raft areas is greater than 50%, cholesterol may precipitate as crystals or form cholesterol/cholesterol bilayer areas, reducing transmembrane oxygen diffusion to about 10% that of healthy membranes. In this way, high levels of dietary PUFAs may serve as the driving force for cellular hypoxia in preeclampsia **(Figure 5 and Figure 6)**.

**Figure 7:**
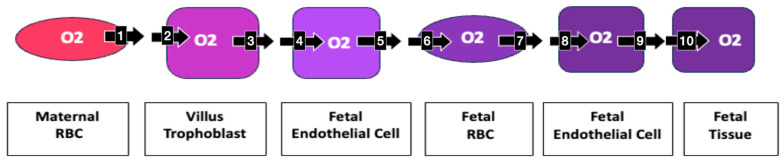
Oxygen’s transplacental path to fetal tissue Moving from a maternal RBC to fetal tissue, an oxygen molecule must pass through 10 membranes: 1. maternal RBC, 2. villous trophoblast maternal lumen side, 3. villous trophoblast stromal side, 4. fetal endothelial cell stromal side, 5. fetal endothelial cell lumen side, 6. fetal RBC at placental villus, 7. fetal RBC at fetal endothelial cell, 8. lumen side fetal endothelial cell, 9. tissue side fetal endothelial cell, 10. fetal tissue cell. Increased impedance at each membrane may become clinically relevant in creating the measured increased oxygenation of maternal villous blood and decreased fetal oxygenation.

For an oxygen molecule to move from a maternal RBC to fetal tissue, it must pass through ten membranes. Increased impedance to the diffusion of oxygen at each membrane helps to explain the increased oxygen of maternal blood in the villous space, as well as the hypoxia of fetal blood **(Figure 7)**.

## 10. Dyslipidemia: Increased Total Cholesterol, Reduced Cholesterol-Dependent Signaling

The etiologic role of cholesterol in preeclampsia is enigmatic. At the systemic level, preeclampsia is associated with dyslipidemia and increased cholesterol in blood lipids, macrophages laden with esterified cholesterol, cholesterol crystals, increased oxysterols, and increased endothelial rafts with a high cholesterol content; murine models of preeclampsia can be created by feeding cholesterol at supraphysiologic levels. Yet at the membrane signaling level, cholesterol deficiency is evidenced not only by reduced cholesterol-dependent signaling processes, such as Hh, Wnt/βcatenin, and eNOS, but also by increased vascular permeability, a reduction in the formation of primary cilia, and increased maternal cholesterol synthesis [105].

### 10.1. Accessible Membrane Cholesterol

The key to this paradox lies in distinguishing free or **accessible membrane cholesterol** available for signaling from **sequestered or inaccessible cholesterol,** which is esterified, oxidized, crystalized, or complexed with other molecules rendering it unavailable for signaling or as an antioxidant [106]. In preeclampsia, six mechanisms may increase sequestration and reduce maternal membrane cholesterol accessibility: (1) increased lipid raft formation, (2) cholesterol crystallization, (3) increased TUFAs, (4) increased oxidative stress with cholesterol serving as an antioxidant by forming oxysterols to reduce free radical propagation, (5) increased cholesterol esters in which oxidized or damaged fatty acids are esterified to cholesterol for removal from cells, and (6) the movement of cholesterol to the fetus and placenta [107,108].

By increasing raft formation, the incorporation of PUFAs and/or LCSFAs into tissues increases the maternal systemic and placental demands for cholesterol. As outlined in Section 5 and Section 6, raft formation is increased by the high melting point disparity of membrane lipids caused by either excessive dietary low-melting-point lipids, such as PUFAs, or high-melting-point lipids, such as LCSFAs. The extremely low cholesterol affinity of PUFAs makes it a major driving force in raft formation. Increased PUFA also leads to increased oxidative stress as discussed in Section 10. Environmental molecules, such as PFAS and PCB, which change membrane viscosity, may also drive this process and are associated with abnormal cholesterol homeostasis in humans [109,110].

With PUFAs driving an increase in high-cholesterol raft areas and the incorporation of high-cholesterol-affinity TUFAs into rafts, localized cholesterol concentrations may rise dramatically causing the precipitation of cholesterol crystals. Studies of gall stone formation link increased unsaturation in phosphatidylcholine to the nucleation and growth of cholesterol crystals at lower cholesterol concentrations [111]. This mechanism may also account for the elevated levels of cholesterol crystals found in lipid droplets typical of cardiovascular disease and nonalcoholic steatohepatitis [112]. Presumably, cholesterol precipitated in crystals is not available for membrane signaling [105]. By increasing lipid rafts, the sequestration of cholesterol in cell membranes, promoting cholesterol crystallization, and increasing cholesterol oxidation and esterification, **dietary PUFAs, LCSFAs, and TUFAs may cause the reduction in accessible cholesterol that is evident in preeclampsia.**

Cholesterol is transferred from maternal circulation to the placenta, released from the placenta to the umbilical circulation, and taken up by the fetus, with fetal cholesterol uptake independent of maternal cholesterol levels [108]. Early primate studies show that around 42% of fetal serum cholesterol originates from the maternal circulation with a strong net cholesterol flux from the mother to fetus. The transplacental transfer of cholesterol from the mother to fetus reduces maternal-accessible membrane cholesterol and may partially explain the rapid resolution of preeclampsia following delivery.

### 10.2. Hedgehog Signaling in Preeclampsia

With normal placental development, there is the deep migration of placental cytotrophoblasts into maternal uterine spiral arteries with extensive endothelialization creating low-resistance vascular sinuses and a broad interface between the separate maternal and fetal circulations. In contrast, in preeclampsia, placental development is compromised by the failure of cytotrophoblasts to transform from a proliferative epithelial subtype into an invasive endothelial subtype causing maternal vessels to remain narrow with a reduced placental connection [1] and leading to placental hypoxia.

The control of placental development lies in a complex balance of angiogenic, antiangiogenic, and apoptotic factors with Vascular Endothelial Growth Factor (VEGF) as the nexus for a cascade of signaling [113]. Activation of the VEGF pathway results in cell division, migration, angiogenesis, and embryonic development. Positioned upstream of VEGF, Hedgehog (Hh) signaling is a key regulator of embryonic and placental development. Both VEGF and Hh signaling localize to projections from maternal endothelial cells called primary cilia, which in preeclampsia, are shorter and fewer in number [114], with diminished Hh signaling [115]. Placental Hh signaling (downregulated in preeclampsia) is highly correlated with the fetal birth weight in murine models [116]. With Hh so intimately involved in all forms of animal growth and development, finding the mechanism controlling Hh signaling has been a top research goal for decades. Surprisingly, highly abundant cholesterol is the primary regulator of Hh signaling. However, it is not total cholesterol, but the availability of **free or accessible membrane cholesterol,** that positively regulates Hh signaling [117,118]. Low accessible cholesterol may also explain the shortening, reduction in number, and impaired function of maternal endothelial cell cilia [87].

### 10.3. Wnt/βcatenin Signaling

Acting on cell proliferation, migration, adhesion, and survival, Wnt/βcatenin signaling is also critical for mediating angiogenesis [113]. In preeclampsia, abnormalities in Wnt/βcatenin signaling lead to severe defects in placental development [119,120]. Wnt/βcatenin signaling may be divided into a canonical pathway, which promotes proliferation and growth and a noncanonical pathway, which promotes apoptosis, a critical factor in the reduced placental development associated with preeclampsia [121]. In Wnt/βcatenin signaling, accessible cholesterol switches signaling from noncanonical to canonical pathways [122]. In this way, a deficit of accessible cholesterol promotes trophoblast apoptosis, while adequate accessible cholesterol promotes angiogenesis.

### 10.4. Endothelial Nitric Oxide Synthase Signaling

Synthesized from L-arginine in a reaction catalyzed by nitric oxide synthases, the soluble gas nitric oxide (NO), plays a role in regulating inflammation, oxidative stress, and vasodilation. Most studies show reduced NO activity in preeclampsia with diminished peripheral endothelial vasodilation and endothelial nitric oxide synthase (NOS3) activity [123] to which oxidative stress contributes [124]. Primarily located in caveolae [125], a type of membrane raft, NOS3 activity is eradicated with the application of methyl-β-cyclodextrin (MβCD), which selectively extracts cholesterol from the plasma membrane. After MβCD treatment, electron microscopy shows that caveolae are no longer present, yet both caveolae and NOS3 activity are restored with the addition of cholesterol [126]. In murine models, statins may restore NOS3 activity by reducing the expression of caveolin-1, an NOS3 inhibitor [127]; in human monocytes, statins redistribute cholesterol from non-caveolar areas of the cell membrane to caveolae [128]. The treatment of caveolae with oxidized LDL also eradicates NOS3 activity, which can be restored by cholesterol treatment [129,130,131]. Studies of mice with absent NOS3 function show an exaggerated response to VEGF-blocking factors with increased hypertension, hepatic dysfunction, thrombocytopenia, and renal dysfunction [132], all symptoms of human preeclampsia.

Each of these critical signaling pathways is downregulated in preeclampsia. A deficiency of accessible membrane cholesterol induced by dietary lipid changes may cause this deficit.

### 10.5. Dyslipidemia and Accessible Cholesterol

In a reductionist view of lipoproteins, Low-Density Lipoprotein (LDL) carries cholesterol from its site of synthesis in the liver to peripheral tissues where LDL receptors (LDLR) are produced by cells in response to deficient cholesterol. HDL cholesterol carries excess cholesterol from peripheral cells to the liver. Patients with the highest levels of LDL cholesterol and the lowest levels of HDL cholesterol are at an increased risk of preeclampsia and CVD. Because cells with a cholesterol deficit are not able to produce an excess of cholesterol for export to HDL, they exhibit reduced cellular cholesterol efflux and increased cholesterol synthesis. Increased cholesterol synthesis and the upregulation of LDLR are considered “pathologic” or “inappropriate” in CVD and preeclampsia because the total cholesterol content of peripheral cells, including endothelial cells, is elevated. However, because the regulation of cholesterol synthesis and LDLR formation are dependent on accessible membrane cholesterol, rather than total cholesterol, the upregulation of cholesterol and LDLR synthesis is expectable with increased sequestered cholesterol and a deficiency in free cholesterol, as occurs in diets high in PUFAs, TUFAs, or LCSFAs.

Statins, medications prescribed for the treatment of hypercholesterolemia, are marketed as inhibitors of the hydroxymethylglutaryl-CoA reductase (HMG-CoAR) enzyme, a rate limiting step in cholesterol synthesis. Statins are effective in lowering total cholesterol, LDL, and triglyceride concentrations while increasing HDL concentrations. Pleiomorphic effects include immunomodulatory, antioxidant, anti-inflammatory, plaque stabilization, and reduction of platelet aggregation [133]. Although blocking HMG-CoAR implies a reduction in cholesterol production, early cholesterol turnover studies with long-term lovastatin therapy showed increased expression of HMG-CoAR, as well as the LDL receptor, and no significant change in total body exchangeable cholesterol [134]. In mouse studies, statins robustly increase cholesterol synthesis primarily in the liver, stimulate fecal cholesterol elimination, increase the expression of sterol regulatory element binding protein 2 (SREBP2, the transcription factor regulating cholesterol synthesis), and strongly induce the gene expression of cholesterol synthesis enzymes with an increase in total body cholesterol synthesis [135]. A study on the effect of dietary cholesterol found that at daily cholesterol intakes less than 900 mg, the concentration of LDL cholesterol increased in the plasma, while the elevation of HDL-C occurred only in the range from 650 to 900 mg/day and not when below 650 mg/day [136]. That is, reduced dietary cholesterol **increases dyslipidemia**. Other studies show that a high intake of eggs, one of the most concentrated dietary sources of cholesterol, is associated with beneficial serum lipid profiles [137]. Oxysterols, which are formed by the oxidation of cholesterol and are much more potent than cholesterol in downregulating cholesterol synthesis, are reduced by statins [138]. Statins also increase serum monounsaturated fatty acids and **reduce serum PUFAs** [139]. These studies raise the possibility that the mode of action of statins is by increasing accessible cholesterol by reducing cholesterol sequestration in membrane rafts, rather than by inhibiting cholesterol synthesis. By reducing serum PUFAs, statins also reduce raft formation and oxidative stress. With adequate accessible cholesterol available to esterify damaged (oxidized) fatty acids for removal from endothelial membranes and transport to the liver for disposal, membrane homeoviscosity may normalize.

## 11. Dietary Lipids and Oxidative Stress

Defined as an imbalance between oxidants and antioxidants, oxidative stress has long been recognized as a major element in preeclampsia pathology [140,141,142]. Oxidative stress occurs when free radicals, molecules containing an unpaired electron, such as reactive oxygen and nitrogen species, interact with biological molecules causing damage. Some exogenous origins of free radicals are ultraviolet radiation, ionizing radiation, and heavy metals [143], with the primary endogenous source being the electron transport chain in mitochondria [144]. Because the surrounding lipid environment may markedly change a protein’s shape and function [145,146], variations in dietary lipids may alter mitochondrial lipids [147] causing conformational shifts in the structure of membrane proteins [148,149,150]. In this way, diet-induced changes in lipids may distort electron transport proteins allowing the escape of unpaired electrons and increasing oxidative stress.

PUFAs, being much more vulnerable to oxidation than monounsaturated fatty acids or saturated fatty acids, markedly increase lipid, protein, and mitochondrial DNA oxidative damage [151]. Like the proliferation of nuclear fission in an atomic reactor, after absorbing a free radical, PUFAs release more free radicals and propagate oxidation [152]. By absorbing a free radical without propagation, antioxidants, such as glutathione peroxidase, superoxide dismutase, coenzyme Q10, selenium, vitamin C, Vitamin E, and **cholesterol** [153,154,155], slow the production of reactive species.

The biological consequences of reactive oxygen species include many pathways active in preeclampsia pathology including the following: (1) activation of redox-sensitive transcription factors, such as NFKβ, COX-2, AP-1, and interleukin 1β, which regulate gene transcription in response to cytokines, growth factors, stress, and infections and also regulate inflammation, immunity, differentiation, proliferation, growth, and survival; (2) increased TNF-α, an inflammatory cytokine; (3) the activation of protein kinases, such as MAPK, SAPK-JNK, and ASK1, which coordinate cell proliferation, differentiation, mobility, and survival; (4) increased levels of the soluble receptor for vascular endothelial growth factor (sFlt-1), which blocks the action of VEGF; (5) increased VEGF; (6) loss of intracellular calcium homeostasis with increased unfolded protein response, endoplasmic reticulum, and mitochondrial stress and apoptosis; (7) lipid-peroxidation-induced change in membrane fluidity and function; (8) oxidation of proteins with both loss and gain of function and misfolding; and (9) nuclear and mitochondrial DNA damage [156,157,158].

The relationship between increased oxidative stress and preeclampsia has been noted for decades, yet the fundamental cause of the imbalance between antioxidants and free radicals remains unclear. Clinical trials using varied antioxidants to treat preeclampsia show no reduction in preeclampsia or preterm birth [159,160,161], leading a recent research team to ask, “Antioxidants in Pregnancy: Do We Really Need More Trials?” [162]. Studies of antioxidants in maternal blood are inconsistent with findings of either reduced or increased antioxidant levels [15,163]. It appears that excess oxidative stress in preeclampsia arises from excess reactive oxygen and nitrogen species, which may be generated from excess PUFAs, rather than primarily from a deficiency in antioxidants.

Because oxysterols more strongly inhibit Hydroxymethylglutaryl-CoA reductase than does cholesterol, the presence of increased oxidative stress may reduce cholesterol synthesis and accessible cholesterol. Oxidized lipids also behave differently in cell membranes, with oxysterols and oxidized PUFAs producing a thinner and more fluid membrane, which further reduces the movement of oxygen across the membrane when compared to unoxidized molecules.

## 12. Lipids in Preeclampsia Screening and Epidemiology

Several factors are widely recognized as increasing the risk of developing preeclampsia, including previous preeclampsia, chronic hypertension, pregestational diabetes, multiple gestation, pre-pregnancy BMI > 30, and antiphospholipid syndrome, with a lesser increased relative risk associated with a history of lupus erythematosus, stillbirth, pre-pregnancy BMI > 25, nulliparity, prior placental abruption, assisted reproductive technology, paternal factors [164], chronic kidney disease, advanced maternal age, and genetic susceptibility [1]. Although less generally noted, abnormal maternal serum lipids also convey an increased risk of preeclampsia, with reduced maternal serum omega-3 fatty acids increasing the relative risk 7.6 times, [5,165,166] elevated maternal red blood cell (RBC) TUFAs increasing the relative risk 3–7.4 times [3,167], and a high dietary intake of total PUFAs increasing the relative risk 2.6 to 5 times [4,14]. Lipidomic studies reflect this pattern with placentae from affected women containing elevated levels of TUFAs [168] and total PUFAs [10,20]. Although there are exceptions [169], several studies show that dietary TUFA intake is positively associated with preeclampsia incidence [18,170] **(Table 1)**.

Membrane-forming lipids, such as phospholipids, ceramides, and sphingomyelins have significantly different patterns in maternal and placental tissues in patients with preeclampsia versus controls [9,10,16,171,172,173]. This marked dysregulation of lipids supports their use in screening for preeclampsia [174,175]. A 2021 pilot study assessing the leptin/ceramide ratio outperformed the more established sFlt-1/PlGF ratio in predicting preeclampsia in terms of detection and sensitivity [176]. Maternal serum metabolic biomarkers, including sphingolipids in mid trimester, also performed better than the sFlt-1/PlGF ratio in screening [177].

## 13. Diets and Preeclampsia

### 13.1. Diets Affect Preeclampsia Incidence

Because biologic membranes adapt their lipid composition in response to dietary lipids [178], dietary lipid variance may be the basis of lipidomic changes in preeclampsia. The important role of the diet was suggested by a 2023 meta-analysis of the effects of a high Dietary Inflammatory Index (DII), which indicated that each 1 unit increase in the DII score is associated with a 24% higher risk of preeclampsia [179]. A 2001 study from Norway showed that a high dietary intake of energy, sucrose, and PUFAs is associated with an increased risk of preeclampsia [4]. A 2023 study of 106,808 pregnant US military personnel without preeclampsia risk factors on a standard military diet with 7% of the total caloric intake as PUFAs and higher PUFAs for those at a high risk of CVD showed a preeclampsia rate of 9.5 to 12% in all ethnic groups [180] compared to a 3 to 8% rate in civilian populations. The Danish National Birth Cohort evaluated 66,738 pregnancies from 1996–2002 of which 0.03% were vegans (*n* = 18). The rate of preeclampsia in omnivores was 2.6%, but it was 11.1% in vegans [19]. A meta-analysis of the effect of the Mediterranean Diet showed a reduction in preeclampsia risk with adherence to the Mediterranean Diet [181]. These studies emphasize the crucial role that diet may play in preeclampsia pathology and the need to consider the role of dietary lipids in metabolic pathways, screening, and treatment.

### 13.2. Increased Dietary PUFAs Increase Preeclampsia Incidence

Metabolic studies suggest a relationship between an increased unsaturation index and preeclampsia. A 2005 study measuring the urinary excretion of isoprostane, an indicator of oxidative damage to lipids, found that increased dietary fat and PUFAs correlate with increased urinary isoprostane excretion and a five-fold increase in preeclampsia risk [182]. A 2021 study from Indonesia, which has one of the world’s highest preeclampsia rates, shows a positive correlation between maternal serum PUFAs and preeclampsia incidence. Preeclampsia was associated with elevated total PUFAs, reduced DHA, a significantly higher omega-6:3 ratio, and a lower omega-3 index compared to controls [20]. Fatty acid desaturases, which are the rate limiting steps in the metabolism of PUFAs, are higher in maternal RBCs of those with preeclampsia compared to controls [183].

### 13.3. Dietary Intervention Studies

Although prospective studies of dietary lipid manipulation in pregnancy are uncommon, available studies are encouraging. In a group of 800 subjects, a 2020 randomized controlled study reducing dietary trans fatty acids showed a reduction in TUFAs between study groups, which was accompanied by a significant reduction in preeclampsia risk (OR 0.56) [18]. A Norwegian study of probiotic milk intake in late pregnancy was significantly associated with a lower preeclampsia risk (OR 0.80) [184]. A meta-analysis of nine studies using olive oil supplementation also showed a reduced risk of preeclampsia [185]. Increased dietary fiber has consistently shown a reduced preeclampsia risk [186] perhaps through the mechanism of improving gut dysbiosis and increasing the production of short-chain saturated fatty acids [187,188,189]. Supplementation with omega-3 fatty acids has had mixed results, with initial studies showing no benefit, but a 2018 Cochran meta-analysis showed the possible reduction of preeclampsia risk with a relative risk of 0.84 in the general population with low-quality evidence, but significant protection among women with a high risk [190]. Weight-management interventions in pregnancy also reduce preeclampsia risk, with a meta-analysis of 88 studies showing a significant decrease in the risk of preeclampsia (RR 0.67) [17]. However, in a population with primarily a low-fat, high-carbohydrate diet likely deficient in choline, supplementation with PUFAs **reduced** the preeclampsia relative risk to 0.40 [21]. Although the Effect of Simple, Targeted Diet in Pregnant Women With Metabolic Risk Factors on Pregnancy Outcomes (ESTEEM) trial treating women with metabolic risk factors with a Mediterranean-style diet showed no reduction in preeclampsia [191], a similar trial, including enhanced extra virgin olive oil and pistachios, in addition to the Mediterranean Diet, did reduce composite maternal and fetal complications, including preeclampsia, although the target for olive oil intake was not met [192]. Overall, these studies suggest that a combination strategy to reduce TUFAs, PUFAs, and the omega 6:3 ratio may be an effective strategy to reduce preeclampsia incidence.

### 13.4. Choline Deficiency and Preeclampsia

Choline is an essential factor, which may be synthesized in small quantities endogenously from phosphatidylethanolamine, although genetic polymorphisms interfere with synthesis. To prevent deficiency, it must be obtained exogenously [193]. With the best dietary sources of choline being animal-based foods, choline deficiency occurs more frequently in vegans. The National Health and Nutrition Examination Survey (NHANES) in the United States indicates that fewer than 11% of pregnant people have adequate intake [194,195]. During pregnancy, choline requirements rise as choline is passed from maternal to fetal tissues with human placentae containing around 50 times more choline than maternal blood [196]. Those with choline deficiency who are not pregnant exhibit preeclampsia-like symptoms with liver changes, such as hepato-steatosis with elevated transaminases [193,197], renal cortical necrosis with lipid accumulation and the reduced reabsorption of water [198], and cardiovascular effects, such as hypertension [199].

Choline is necessary for the synthesis of phosphatidylcholine, as well as sphingomyelin. Given a choline-deficient diet with a high carbohydrate intake, the fewer phospholipid headgroups available to esterify fatty acids may cause the lengthening of fatty acid chains and an increase in LCSFAs. Rodent models using a choline-deficient diet confirm this analysis, with lipidomics showing major reductions in serum phospholipids and sphingolipids, which are restored after treatment with phosphatidylcholine [200].

### 13.5. Preeclampsia with PUFA Deficiency

Although many studies implicate the involvement of excessive PUFAs in increased preeclampsia risk, dietary studies in populations with much different diets than those found in the United States indicate the potential involvement of LCSFAs and/or choline deficiency. For example, in low-to-moderate income populations with a baseline high carbohydrate, low protein, low PUFA diet, supplementation with PUFAs significantly reduces the relative risk of preeclampsia [21]. In another population with a similar diet, the use of a lipid-based high-PUFA nutritional supplement in underweight women diagnosed with preeclampsia was found to lead to significant improvements in birthweight, gestational age at delivery, head circumference, and birth length [201]. To understand how these dietary interventions fit with the lipid raft model of preeclampsia outlined in this review, consideration must be given to the effects of excessive LCSFAs with deficient PUFAs and choline on membrane biophysics.

Returning to Figure 3, which illustrates homeoviscous adaptation with an excessively fluid lipid bilayer, the Levental experiments show the essential compensatory action of adding LCSFAs and cholesterol to return the membrane to a normal viscosity. When choline and PUFA deficiency create an excess of LCSFAs, as may be found in high-carbohydrate/low-protein diets, membrane lipid bilayers may be excessively stiff. In this setting, the addition of PUFAs in the form of essential fatty acids increases fluidity and restores membrane homeoviscosity. With a large membrane melting point disparity, lipid rafts and cholesterol sequestration are increased, creating preeclampsia without prominent elements of oxidative stress. From these studies, we may begin to suspect that the critical factor in the etiology of preeclampsia may be the balance of low-, moderate-, and high-melting-point lipids and cholesterol, rather than absolute values or deficiencies.

### 13.6. Dietary Cholesterol

Since the 1970s when the United States Department of Agriculture first published guidelines recommending the limitation of dietary cholesterol to reduce cardiovascular disease until these guidelines were withdrawn in 2015, the role of dietary cholesterol in the causation of CVD has been vigorously debated. More recent analyses not only confirm the absence of harm, but also show the beneficial effects of dietary cholesterol challenges on human plasma lipoprotein subfractions [202]. In preeclampsia, one study shows a statistically significant **lower** dietary cholesterol intake in human subjects with preeclampsia (361.48 mg/day) versus controls (407.88 mg/day) [22]. A source of confusion in the role of cholesterol in dietary studies may arise from dietary questionnaires, which frequently group together dietary cholesterol, oxysterols, long-chain saturated fatty acids, and trans fatty acids under the category of a “Western Style Diet”, which is associated with an increased preeclampsia incidence [203]; thus, dietary cholesterol is condemned by association rather than causation.

## 14. Discussion

The consideration of the role of lipids in preeclampsia pathology presents four major goals for lipid therapy:

1. Reduce oxidative stress: With very-low-melting-point PUFA as the driving force for oxidative stress, replacing dietary PUFAs with medium-chain saturated fatty acids, such as lauric acid (C12:0) in coconut oil, monounsaturated fatty acids, such as oleic acid (C18:1) in olive oil, or short-chain saturated fatty acids, such as butyric (C2-5:0), which are found in milk products or as products of gut bacteria, should markedly reduce oxidative stress, as these lipids are less vulnerable to oxidative damage. Avoiding dietary oxysterols, which are found in dehydrated milk, eggs, and proteins, also reduces oxidative stress [204]. These measures should lower blood pressure, restore normal angiogenic signaling, and reduce protein misfolding.
2. Reduce lipid raft formation and hypoxia: Lowering dietary PUFAs reduces the disparity in membrane lipid melting points and the need for endogenous LCSFA synthesis, thus lessening lipid raft formation and hypoxia. A reduction in membrane PUFAs also reduces the formation of cholesterol crystals and cholesterol/cholesterol bilayer domains, which markedly impair the movement of oxygen across placental membranes. LCSFAs, such as palmitoleic (C:16) and stearic acid (C18:0) found in beef, pork, lamb, and chicken, may also stimulate excessive lipid raft formation, and are elevated in some with preeclampsia [205,206], so intake should be low or moderate. Supplementation with moderate-melting-point fatty acids to inhibit raft formation and with choline in those who are deficient should be beneficial [103].
3. Increase accessible membrane cholesterol: Increasing dietary cholesterol allows for esterification and the removal of damaged fatty acids, as well as reductions in oxidative stress. Reducing dietary TUFAs by avoiding foods cooked at high temperature in oil and meats produced with feeds using spent deep fryer oils should reduce cholesterol sequestration in rafts and cholesterol esters. Supplementing with dietary cholesterol provides accessible cholesterol to restore normal Wnt, Hh, and eNOS signaling, aids in angiogenesis, improves vasodilation, and restores normal membrane rheology and permeability.
4. Reduce the omega 6:3 ratio to less than 4:1: Monitoring the omega 6:3 ratio allows for the identification of cases in which an elevated 6:3 ratio (>4:1) is clinically significant and whether supplementation has been effective. This measure should reduce inflammation and thrombosis.

Strategies to meet the goals of reducing oxidative stress, hypoxia, lipid raft formation, and cholesterol sequestration and optimizing the omega 6:3 ratio can be met over long periods of time through diets high in fruits and vegetables with the reduced intake of total fats, PUFAs, sugar, processed and high-fat meats, deep-fried foods, oxysterols, and excess calories [207]. Both the Mediterranean Diet and the New Nordic Diet have been effective in reducing preeclampsia. Ideally, months before conception, a person contemplating pregnancy would begin an optimal diet to reduce the risk of preeclampsia. However, dietary change is difficult, as evidenced by the high rate of obesity in the United States, and modified cooking techniques have not yet been widely incorporated into dietary advice. The expansion of previous successful clinical trials to reduce preeclampsia, which are aimed at reducing trans fatty acids, improving the omega 6:3 balance, and reducing excessive PUFAs in those contemplating pregnancy, provides a starting point to prevent preeclampsia.

Because dietary lipids are incorporated into endothelial cell membranes within hours of ingestion, there is reason for optimism that lipid-rescue therapy for mothers hospitalized with preeclampsia may affect a patient’s status within a relatively short, clinically relevant time. With the volume of lipids in maternal endothelial cells, the placenta, and transferred to the fetus increasing as gestation advances and because very premature deliveries carry higher fetal risks, perhaps the initial proof-of-concept protocol should target early preterm (<30 weeks) gestations, where lipid treatment is most likely to be successful.

## 15. Conclusions

This review explores the role that dietary lipids may play in creating placental hypoxia and reducing accessible membrane cholesterol and cholesterol-dependent signaling. By changing membrane viscosity and raft formation, dietary lipids, environmental factors, such as inhaled hydrocarbons, and membrane viscosity modifiers, such as PFAS may reduce the movement of oxygen across membranes, sequester cholesterol, and modify raft-related signaling. Metabolomic, metabolic, and interventional studies demonstrate close correlations among dietary lipid intake, environmental membrane modulators, and preeclampsia risk. The encouraging results of interventional trials merit further investigation of the use of dietary lipids to prevent and treat preeclampsia.

## Figures and Tables

**Table 1:** **Preeclampsia risk factors.** In addition to standard preeclampsia risk factors, such as chronic hypertension, pregestational diabetes, multiple gestation, BMI > 30, and antiphospholipid antibodies, lipid-related factors, such as elevated PUFAs and TUFAs and low omega 3 fatty acids also confer a substantial risk.

Condition	Relative Risk
Previous pregnancy with preeclampsia	8.4
Chronic hypertension	5.1
Pregestational Diabetes	3.7
Multiple gestation	2.9
Pre-pregnancy BMI > 30	2.8
Anti-phospholipid Antibodies	2.8
Reduced maternal serum omega-3 fatty acids	7.6
Increased maternal RBC TUFA	3 to 7.4
Increased dietary PUFA	2.6 to 5
